# Dioscin strengthens the efficiency of adriamycin in MCF-7 and MCF-7/ADR cells through autophagy induction: More than just down-regulation of MDR1

**DOI:** 10.1038/srep28403

**Published:** 2016-06-22

**Authors:** Changyuan Wang, Xiaokui Huo, Lijuan Wang, Qiang Meng, Zhihao Liu, Qi Liu, Huijun Sun, Pengyuan Sun, Jinyong Peng, Kexin Liu

**Affiliations:** 1 Department of Clinical Pharmacology, College of Pharmacy, Dalian Medical University, Dalian, Liaoning, China; 2 Provincial Key Laboratory for Pharmacokinetics and Transport, Liaoning, Dalian Medical University, Dalian, Liaoning, China

## Abstract

The purpose of present study was to investigate the effect of dioscin on activity of adriamycin (ADR) in ADR-sensitive (MCF-7) and ADR-resistant (MCF-7/ADR) human breast cancer cells and to clarify the molecular mechanisms involved. Antiproliferation effect of ADR was enhanced by dioscin in MCF-7 and MCF-7/ADR cells. Dioscin significantly inhibited MDR1 mRNA and protein expression and MDR1 promoter and nuclear factor κ-B (NF-κB) activity in MCF-7/ADR cells. Additionally, inhibitor κB-α (IκB-α) degradation was inhibited by dioscin. Moreover, dioscin induced the formation of vacuoles in the cytoplasm and protein level of LC3-II in MCF-7 and MCF-7/ADR cells. Autophagy inhibitor 3-MA abolished the effect of dioscin on ADR cytotoxicity. Dioscin inhibited phosphorylation of PI3K and Akt, resulting in upregulation of LC3-II expression. In conclusion, dioscin increased ADR chemosensitivity by down-regulating MDR1 expression through NF-κB signaling inhibition in MCF-7/ADR cells. Autophagy was induced by dioscin to ameliorate the cytotoxicity of ADR via inhibition of the PI3K/AKT pathways in MCF-7 and MCF-7/ADR cells. These findings provide evidence in support of further investigation into the clinical application of dioscin as a chemotherapy adjuvant.

Breast cancer is the most common cancer and the leading cause of cancer death among females worldwide, with an estimated 1.7 million cases and 521,900 deaths in 2012^1^. It is estimated more than 235,000 invasive breast cancer patients with 40,430 deaths in the United States in 2014^2^ . Despite scientific and medical breakthroughs in the breast cancer therapy, the survival rates for metastatic breast cancer are currently estimated less than 25% for 5-year and 5–10% for 10-year^3^. Resistance to therapeutic interventions remains to be great challenges in clinical management for breast cancer patients. About 40–50% of these tumors will display *de novo* or acquired resistance, and all patients develop acquired resistance to multiple agents over time^4^. Drug resistance in breast cancer includes chemotherapy resistance, endocrine therapy resistance and HER-2 targeted therapy resistance^5^. Resistance to chemotherapeutic agents, in particular, multi-drug resistance (MDR), is a major cause of treatment failure in cancer^6^. MDR1, a human multidrug resistance transporter first discovered in drug-resistant Chinese hamster ovarian cells, is a 170 kD plasma membrane glycoprotein encoded by the MDR1 gene, and belongs to the ATP binding cassette family^7^,^8^. Although several mechanisms of multidrug resistance have been elucidated, the most common involves the overexpression of MDR1. MDR1 has been extensively studied due to its importance to human tumor. In tumor cells, MDR1 pumps out anticancer drugs, leading to drug resistance at the cellular level^6^,^9^. It plays an important role in producing MDR in breast cancer cells^10^. Studies of multidrug resistance mechanisms have relied on the analysis of cancer cell lines that have been selected and present cross-reactivity to a broad range of anticancer agents. In the current study, adriamycin-sensitive human breast cancer cells (MCF-7) provide a useful *in vitro* model system to study breast cancer. An MCF-7 cell line which was selected for resistance to adriamycin (MCF-7/ADR, MCF-7/adriamycin) exhibits the phenotype of MDR. The MDR in MCF-7/ADR is associated with overexpression of the MDR1 gene product. Currently, many clinical anti-cancer drugs such as certain alkaloids, anthracycline antibiotics, and epipodophyllotoxin derivatives can induce MDR^11^. MDR1 inhibition with MDR reversal agents could potentially attenuate MDR, increasing anticancer drug cytotoxicity^12^. The discovery and development of safe and effective MDR reversal agents is urgently required.

In recent years, researchers have focused on the relationship between autophagy and MDR. Autophagy is a major intracellular degradation process responding to stress conditions to either promote survival during starvation or lead to type II programmed cell death^13^. As a double-edged sword, autophagy may lead to survival of MDR tumors, or its activation may lead to cancer cells’ death^14^. On one hand, autophagy occurs to promote cell survival after exposure of cytotoxic drugs. Combination use of autophagy inhibitors was thought to be a new strategy to overcome MDR^15^,^16^. On the other hand, compounds that induce apoptosis-independent autophagic cell death can be effective against drug resistant tumors, either used alone or in association with conventional chemotherapeutics^17^,^18^. Thus, it is necessary to understand the exact role of autophagy (prodeath or prosurvival) induced by some agent/drug.

Dioscin is an active ingredient of Dioscorea nipponica Makino, a traditional herb medicine commonly used in Asian. Pharmacological research has demonstrated that dioscin has anti-inflammatory, lipid-lowering, anticancer, and hepatoprotective effects^19^,^20^,^21^. Our studies have shown that dioscin restores the activity of the anticancer agent adriamycin (ADR) in MDR human leukemia K562/adriamycin cells and enhances methotrexate absorption by down-regulating MDR1 via a mechanism involving NF-κB signaling inhibition^12^,^22^. Whether dioscin can increase the activity of ADR in breast cancer cells is presently unclear. In human lung cancer cell lines, dioscin induced autophagy in the early stage of apoptosis induced by dioscin, which protected cell and promote cell survival^15^. However, effect of autophagy induced by dioscin on the MDR was not understood. In the current study, MCF-7 and MCF-7/ADR cells were used to study the effect of dioscin on ADR activity. To understand the molecular mechanism involved, the effect of dioscin on MDR1 expression and the relationship between MDR1 expression and NF-κB activity were also investigated in MCF-7 and MCF-7/ADR cells. Moreover, whether diosicn is an autophagic inducer in MCF-7 and MCF-7/ADR cells and the relationship between autophagy and MDR were definitively determined. We found that dioscin synergizes with ADR to decrease viability of not only ADR-resistant but also ADR-sensitive MCF7 cells, which was different from K562 cells in our previous study^12^. Further study revealed that dioscin increased the activity of ADR in MCF-7 and MCF-7/ADR cells at nontoxic concentrations by down-regulating MDR involving the inhibition of the NF-κB signaling pathway in MCF-7/ADR cells, and worked by activation of autophagy via inhibition of PI3K/Akt pathway in both MCF-7 and MCF-7/ADR cells. These findings provided the potential use of dioscin in clinical application as MDR reversal agent.

## Results

### The cytotoxicity of dioscin

**Figure 1:**
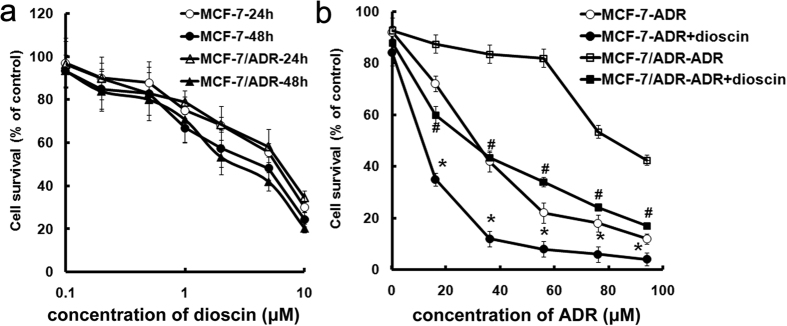
Dioscin at nontoxic concentration increased ADR chemosensitivity in MCF-7 and MCF-7/ADR cells. (**a**) Cells were treated with dioscin (0.1–10 μM) for 24 or 48 h and the survival cells were determined by MTT assay. (**b**) Cells were treated with ADR (0.1–100 μM) with or without dioscin (0.4 μM) for 24 h and the survival cells were determined by MTT assay. *^,#^p < 0.01 versus that obtained in the absence of dioscin.

In order to detect the cytotoxicity of dioscin, we used various concentrations of dioscin (from 0.1 to 10 μM) to determine the cell viability by MTT assay. Both MCF-7 and MCF-7/ADR cells were almost equally sensitive to dioscin, and dioscin exerted toxicity on MCF-7 and MCF-7/ADR cells with IC_50_ of 6.5 ± 0.4 μM and 7.3 ± 0.2 μM, respectively (p > 0.05) (Fig. 1a). These results indicated that dioscin was very effective against both the drug-sensitive parental and multidrug resistance cancer cells. MCF-7/ADR and MCF-7 cells were used to determine the non-toxic concentration of dioscin, namely IC_10_. The concentrations of dioscin IC_10_ in both MCF-7 and MCF-7/ADR cells were 0.4 ± 0.1 μM. Consequently a concentration of 0.4 μM dioscin was chosen for further use in this study.

### Effects of dioscin on ADR toxicity in MCF-7 and MCF-7/ADR cells

To examine whether the IC_10_ of dioscin can affect the cytotoxicity of ADR in MCF-7/ADR and MCF-7 cells, cell viabilities were analyzed by MTT method. The co-incubation with IC_10_ of dioscin and various concentration of ADR synergized the activity of ADR in MCF-7 cells (Fig. 1b). The IC_50_ values in ADR and diosin + ADR were 1.5 ± 0.1 μM and 0.4 ± 0.1 μM in MCF-7 cells, respectively. In turn, IC_10_ of dioscin also remarkably increased cytotoxicity of ADR against MCF-7/ADR cells (Fig. 1b). The IC_50_ values in ADR and diosin + ADR were 34.7 ± 1.1 μM and 0.7 ± 0.1 μM in MCF-7/ADR cells, respectively. Namely, the co-incubation of dioscin and ADR decreased significantly the IC_50_ values of ADR in both MCF-7 and MCF-7/ADR cells compared to ADR alone. These results indicate that dioscin potentiates the cytotoxic effect of ADR in both MCF-7 and MCF-7/ADR cells.

### Effect of dioscin on ADR accumulation in MCF-7 and MCF-7/ADR cells

**Figure 2:**
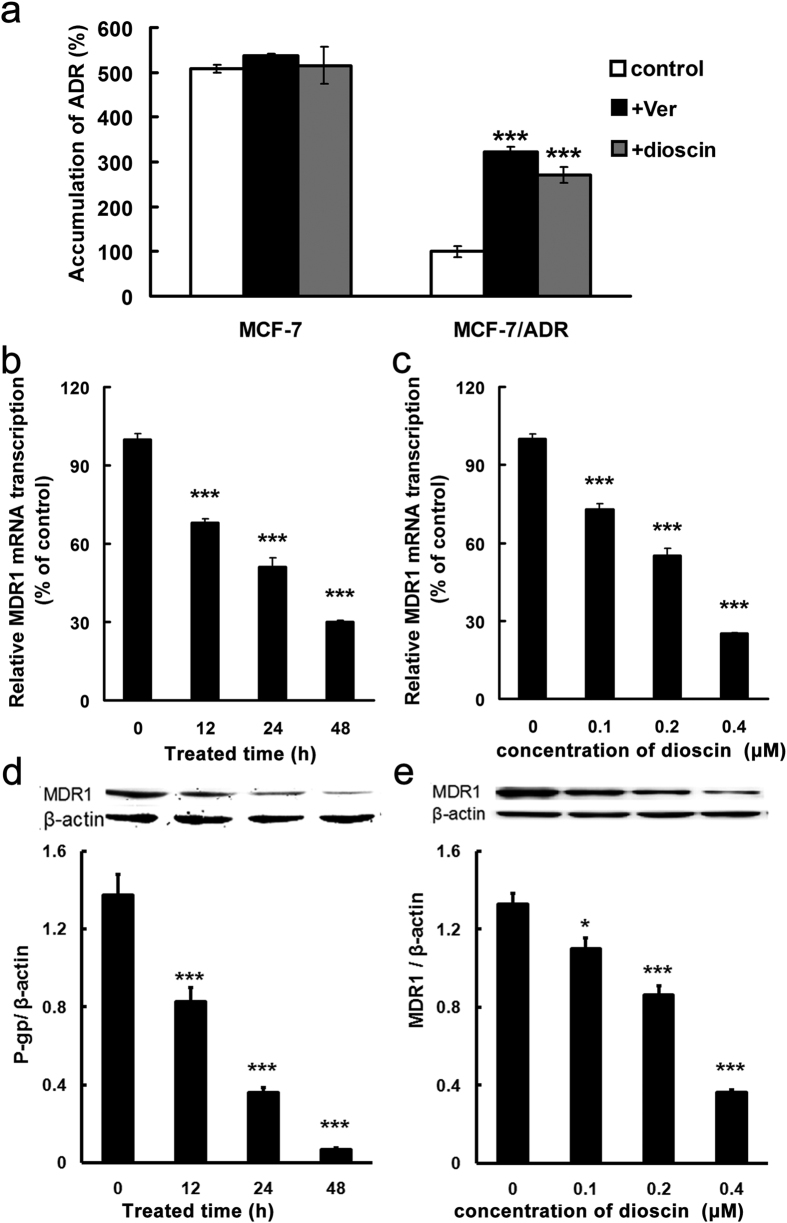
Dioscin inhibited MDR1 expression in MCF-7/ADR cells. (**a**) Cells were pre-incubated with or without dioscin (0.4 μM) or verapamil (Ver, 20 μM) for 24 h and intracellular accumulation of ADR were determined by LC-MS/MS after incubation of ADR for 1 h. (**b–e**) Cells were treated with 0.4 μM dioscin for 0−48 h (**b,d**) or different concentrations of dioscin (from 0 to 0.4 μM) for 48 h (**c,e**). Total RNA was extracted and MDR1 expression was analyzed by qRT-PCR (**b,c**). Lysates of cells were electrophoresed and the expression of MDR1 was detected with an MDR1-specific antibody (**d,e**). The β-actin band is shown to confirm integrity and equal loading of protein. Means ± SD of three experiments are presented. ***p < 0.001 versus that obtained in control group.

To reveal the mechanism involved in sensitization effect of dioscin, intracellular level of ADR was determined in MCF-7 and MCF-7/ADR cells. After an incubation of ADR for 1h, the level of ADR in MCF-7 cells was 4-fold higher than that in MCF-7/ADR cells (Fig. 2a). Incubation of dioscin or MDR1 inhibitor verapamil did not change the intracellular accumulation of ADR in MCF-7 cells, but increased the levels in MCF-7/ADR cells (Fig. 2a). ADR accumulation in verapamil or dioscin treatment group was about three times higher than that in control group in MCF-7/ADR cells (Fig. 2a). The results suggested that inhibition of MDR1 might account for the sensitization effect induced by dioscin in MCF-7/ADR cells. As for MCF-7 cells, some unknown mechanism might be involved in.

### Effect of dioscin on expression of MDR1 mRNA and protein in MCF-7/ADR cells

To investigate the effects of dioscin on MDR1 in MCF-7 and MCF-7/ADR cells, MDR1 gene and protein expression were examined by qRT-PCR and Western blotting. Unlike MCF-7/ADR cells with overexpression of MDR1, level of MDR1 gene and protein expression was too low to be detected in MCF-7 cells. After MCF-7/ADR cells were treated with dioscin, the MDR1 mRNA concentration declined by 69.6% after 48 h (Fig. 2b) and further decreased by 74.8% in concentration of 0.4 μM (Fig. 2c) respectively. The observed changes in MDR1 protein expression in MCF-7/ADR cells were confirmed by Western blot analysis following incubation of the cells for 48 h with various concentrations of dioscin or 0.4 μM of dioscin for the indicated time (0–48 h). The protein level of MDR1 was decreased by 94.3% after 48 h (Fig. 2d) and was decreased by 73.4% in concentration of 0.4 μM (Fig. 2e), respectively. Taken together, these data suggested that dioscin suppresses MDR1 in MCF-7/ADR cells and can reverse ADR resistance.

### The effect of dioscin on NF-κB signaling pathway in MCF-7/ADR cells

**Figure 3:**
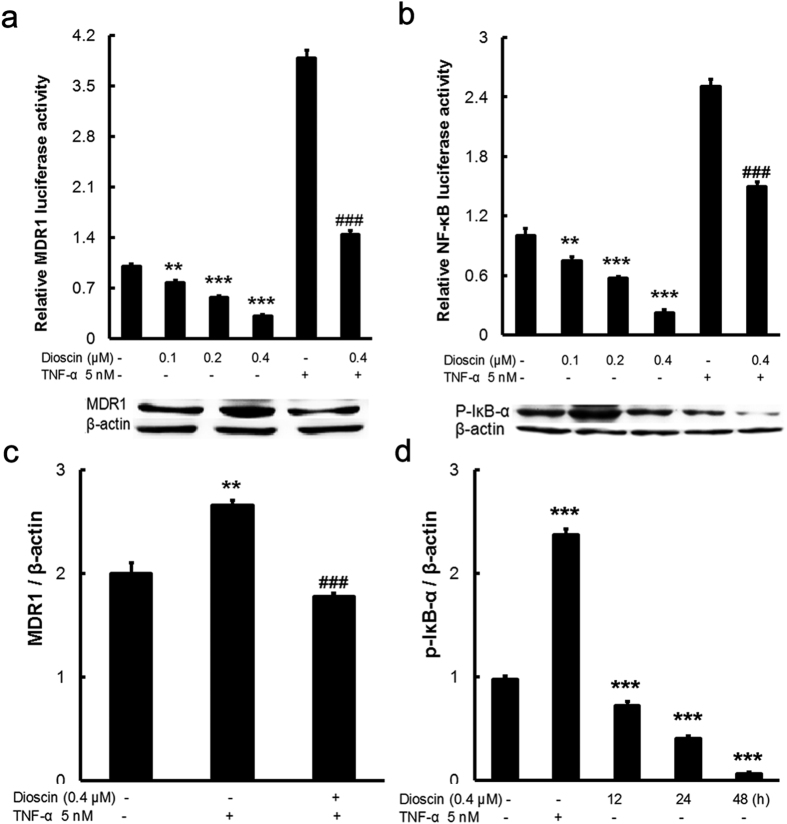
Dioscin down-regulated MDR1 expression via NF-κB signaling inhibition in MCF-7/ADR cells. Cells were transiently transfected with MDR1 (**a**) and NF-κB (**b**) promoter plasmid. After treatment with dioscin and TNF-α, luciferase activity was determined and normalized. (**c**) Effects of dioscin and TNF-α on MDR1 protein expression in MCF-7/ADR cells by Western blotting. (**d**) Effects of dioscin and TNF-α on phospho-IκB-α protein expression in MCF-7/ADR cells by Western blotting. Means ± SD of three experiments are presented. ***p < 0.001 compared to control group; ^###^p < 0.001 compared to TNF-α group.

To elucidate the molecular mechanism for synergized the activity of ADR by dioscin, we detected the effect of dioscin on the MDR1 and NF-κB promoter. The activities of NF-κB and MDR1 promoter were markedly enhanced by TNF-α. The increase in activity caused by TNF-α was sharply inhibited by dioscin (Fig. 3a,b). These results suggest that dioscin inhibits the activity of MDR1, at least in part, by down-regulating NF-κB activity.

Next, to further determine the effect of dioscin on NF-κB signaling pathway, MCF-7/ADR cells were treated with various concentrations of dioscin and TNF-α. Western blot analysis was performed to detect changes in MDR1 and IκB-α protein expression. MDR1 expression was increased by TNF-α, but the effect of increase was inhibited significantly by dioscin (Fig. 3c). Furthermore, TNF-α significantly increased the level of phosphorylation of IκB-α, but this effect was decreased by dioscin at 12–48 h (Fig. 3d). Thus, at least in part, dioscin suppresses NF-κB activation through inhibition of phosphorylation of IκB-α and then inhibited the expression of MDR1 in MCF-7/ADR cells.

### Induction of autophagy by dioscin in MCF-7 and MCF-7/ADR cells

**Figure 4:**
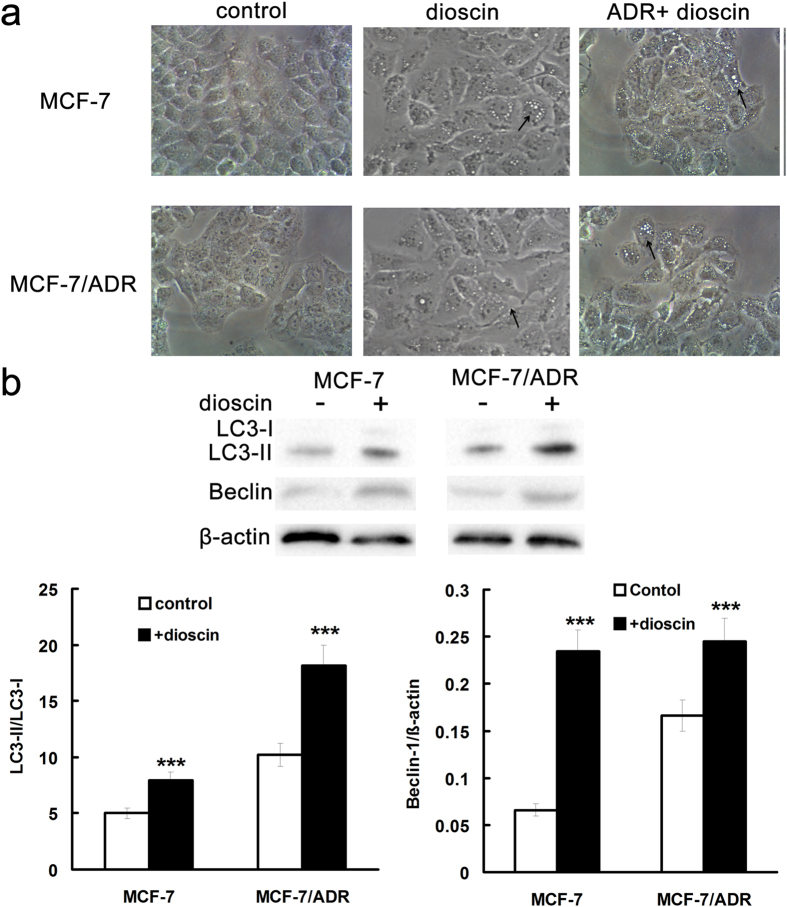
Dioscin induced autophagy in MCF-7 and MCF-7/ADR cells. (**a**) Cells were treated with 0.4 μM dioscin for 24 h and cellular morphology was observed by phase-contrast microscopy. Vacuoles in the cytoplasm were marked by arrowhead. Magnification: 400×. LC3-II and beclin-1 protein expression was determined by Western blotting (**b**). Means ± SD of three experiments are presented.***p < 0.001 versus that obtained in control group.

To understand the mechanism involved in the sensitization effect of dioscin in MCF-7 cells, cellular morphology was observed using microscope. After 24-h treatment with dioscin, besides various numbers of cytoplasmic vacuoles, morphology of sensitive and drug resistant cells was similar as that of control group (Fig. 4a). After treatment with ADR and dioscin, evident signs of cell damage were observed and cells appeared to be shrunken, many of them became round and tended to detach from the substrate (Fig. 4a). The number of cytoplasmic vacuoles increased and the size become bigger than that in dioscin alone group. The morphology changes suggested autophagy induced by dioscin treatment.

To further make clear whether autophagy was induced by dioscin, the protein expression of LC3-I/II and beclin-1, two autophagy makers, were determined by Western blotting. In MCF-7 and MCF-7/ADR cells, LC3-II and beclin-1 protein expression was increased by dioscin compared with corresponding control group, respectively (Fig. 4b). These results suggested that dioscin at concentration of IC10 induced autophagy in MCF-7 and MCF-7/ADR cells. Autophagy induction by dioscin at nontoxic concentration was also observed in MDA-MB-231 cells, another commonly used ER- breast cancer line (See Supplementary data, Fig. S1 and S2).

### Role of autophagy in the sensitization effect of dioscin in MCF-7 and MCF-7/ADR cells

**Figure 5:**
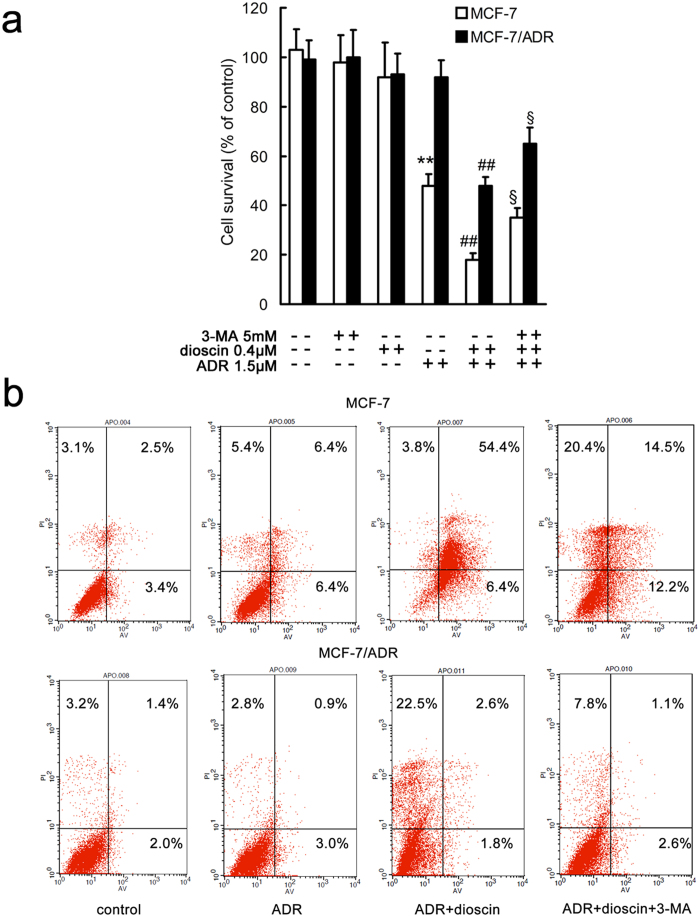
Inhibition of autophagy by 3-MA weakened the sensitization effect of dioscin in MCF-7 and MCF-7/ADR cells. A, cells were treated with 3-MA, dioscin and/or ADR for 24 h and cell viability was determined by MTT assay (**a**) or Annexin V/PI double staining (**b**). **p < 0.01 versus that obtained in the corresponding control group. ##p < 0.01 versus that obtained in the corresponding ADR alone group. §p < 0.05 versus that obtained in the corresponding ADR + dioscin group.

To clarify the role of autophagy on dioscin induced sensitization effect, autophagy inhibitor 3-MA was used in MTT assay and apoptosis assay. Incubation with dioscin enhanced the cytotoxic effect of ADR compared with corresponding ADR alone group in MCF-7 and MCF-7/ADR cells (Fig. 5a). The sensitization effect of dioscin was partially abolished by 3-MA in ADR + dioscin + 3-MA group in both sensitive and resistance cells (Fig. 5a). 3-MA (5 mM) or dioscin (0.4 μM) alone had no effect on the cell survival (Fig. 5a). The results suggested that inhibition of autophagy weakened the sensitization effect of dioscin. Autophagy induction might play an important role in the sensitization effect of dioscin in MCF-7 cells.

The results of Annexin V/PI double staining confirmed the above speculation. The fraction of apoptotic cells induced by ADR was significantly increased by dioscin in MCF-7 and MCF-7/ADR cells compared with corresponding ADR alone group (Fig. 5b). When 3-MA was used, dioscin induced enhancement in cell death was decreased (Fig. 5b). These results indicated that autophagy induced by dioscin played a prodeath role in ADR treated MCF-7 and MCF-7/ADR cells. The same results were found in MDA-MB-231 cells (See Supplementary data, Fig. S3).

### PI3K/Akt pathway in the autophagy induced by dioscin in MCF-7 and MCF-7/ADR cells

**Figure 6:**
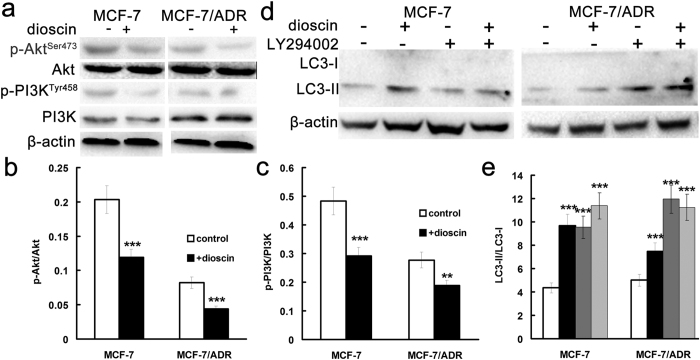
Dioscin induced autophagy through inhibition of PI3K/AKT pathways in MCF-7 and MCF-7/ADR cells. (**a–c**) Effect of dioscin (0.4 μM) on the levels of phosphorylation of PI3K and Akt in MCF-7 and MCF-7/ADR cells. (**d,e**) Effect of dioscin and/or PI3K/AKT inhibitor LY294002 on the levels of LC3 protein expression in MCF-7 and MCF-7/ADR cells. Cells were treated with dioscin (0.4 μM) or LY294002 (20 μM) for 24 h. Cell lysates were prepared and analyzed by Western blot analysis. ***p < 0.001, **p < 0.01 versus that obtained in the corresponding control group.

To finally reveal the signaling pathway involved in the autophagy induced by dioscin, protein expressions of PI3K/Akt pathway and LC3-I/II were detected after treatment of dioscin and/or LY294002 in MCF-7 and MCF-7/ADR cells. Compared with corresponding control group, the levels of phosphorylated PI3K and Akt were decreased significantly after incubation of dioscin (Fig. 6a–c) in both cell lines. At the same time the expression of PI3K and Akt was unchanged by dioscin (Fig. 6a–c). Moreover, effects of inhibition of PI3K/Akt pathway on the LC3-I/II levels were determined and results exhibited that the levels of LC3-II were up-regulated by dioscin as well as LY294002, an inhibitor of PI3K/Akt pathway (Fig. 6d,e). Similarly, dioscin induced autophagy by inhibition of PI3K/AKT in MDA-MB-231 cells (See Supplementary data, Fig. S4). Taken together, dioscin-induced autophagy is mediated by inhibition of PI3K/AKT.

## Discussion

Treatment of breast cancer is now challenged by *de novo* and acquired resistances to radiation, chemotherapy or targeted therapies^23^,^24^,^25^,^26^. The mechanism by which resistance occurs is still not completely known. While cancer stem cells or cancer-initiating cells may contribute to radiation resistance of breast cancer^26^, endocrine resistance and HER-2 targeted therapy resistance may involve the loss of estrogen receptor (ER) alpha expression^24^ and incomplete blockade of the HER receptors^23^. Moreover, many of the human ABC proteins , such as P-gp, MRP1 and BCRP, have been implicated to be the major efflux transporters responsible for multidrug resistance in all types of cancer including breast cancer^6^. Chemotherapy drugs combined with efflux transporter modulators is an important strategy for reversing MDR in cells expressing ABC transporters. However, unfavorable side effects and toxicity profiles at clinical doses have limited its therapeutic applications. Recently, the use of plant materials as anti-tumor agents has gained a great deal of attention for its possible therapeutic potential^27^. Dioscin is a traditional oriental herbal medicine component and exhibits potent antivirus, immunomodulation and anticancer activities^28^,^29^,^30^. The aim of this study was to evaluate dioscin as an effective and safe MDR reversing agent, to gain insight into the sensitization effect of dioscin and to clarify the underlying molecular mechanism using a MDR breast cancer cell line MCF-7/ADR. In the present study, dioscin was found to increase ADR chemosensitivity through down-regulating MDR1 expression via NF-κB signaling inhibition in MCF-7/ADR cells (Figs 1, 2, 3). Interestingly, sensitization effect of dioscin was observed not only in MCF-7/ADR cells, but also in MCF-7 cells (Fig. 1b), and MDA-MB-231 cells (See Supplementary data, Fig. S1), with low expression level of MDR1^31^, suggesting a MDR1-independtend mechanism. Finally, we found that induction of autophagy by dioscin promoted ADR-induced cytotoxicity in breast cancer lines (Figs 4–6, and Figs S2–S4).

### Dioscin at nontoxic concentration increased ADR chemosensitivity in MCF-7 and MCF-7/ADR cells

Combination of MDR-reversal agent with ADR is a strategy aiming to overcome MDR and enhance the anti-tumor effect^32^,^33^. Puerarin, a natural isoflavonoid from Pueraria lobata, was demonstrated to increase cytotoxicity of ADR in MCF-7/ADR cells^34^. In our previous study, dioscin reversed MDR in K562/ADR cells^12^. These natural products exert efficient MDR reversal activity via down-regulation of MDR1^12^,^34^. In the present study, the combination of dioscin at nontoxic concentration (0.4 μM) and ADR enhanced the cytotoxicity toward both MCF-7 and MCF-7/ADR cells (Fig. 1b). It was worth noting that MCF-7 cells were more sensitive to ADR in with the presence of dioscin (Fig. 1b), which was not reported by others^12^,^34^. Moreover, the intracellular accumulation of ADR in MCF-7 cells was not changed by incubation with dioscin or verapamil (Fig. 2a). Therefore, we speculated that reversal effect of dioscin in MCF-7/ADR cells might be related to inhibition of MDR1 while a MDR1-independtend mechanism would be involved in sensibilization effect of dioscin in MCF-7 cells.

### Dioscin down-regulated MDR1 expression via NF-κB signaling inhibition in MCF-7/ADR cells

MDR1 is overexpressed in MCF-7/ADR cells and such tumors are three times less likely to respond to chemotherapy than those that do not express MDR1^35^. The identification of novel agents which can inhibit expression of MDR1 is of utmost interest in cancer research. Our results indicated that dioscin suppresses the expression of MDR1 in MCF-7/ADR cells and can synergize the activity of ADR (Fig. 2b–e).

Many studies have provided evidence implicating complex mechanisms for transcriptional regulation of the MDR1 gene in human cancer cells^12^,^34^,^36^. The up-regulation of MDR1 gene expression was mentioned to correlate with NF-κB signaling pathway, ERK pathway, Cyclooxygenases-2, protein kinase C^37^,^38^,^39^,^40^. Our data showed that dioscin did not block the phosphorylation of ERK in both MCF-7 and MCF-7/ADR cells (data not shown).

NF-κB signaling pathway is reported most frequently to be the molecular mechanism for up-regulating MDR1 gene expression^39^. Human MDR1 gene promoter contains a recognition sites for NF-κB transcription factors^39^. NF-κB inhibitors can decrease MDR1 protein expression and restore chemosensitivity^41^. TNF-α is a well known pro-inflammatory stimuli and activates IκB kinases (IKK) which phosphorylate IκB and release the active NF-κB dimmers^42^. In present study, TNF-α (5nM) increased the activities of MDR1 and NF-κB promoter, the phosphorylation of IκB-α, as well as the expression of MDR1, but the increasing trends were reversed by dioscin (Fig. 3). Gamabufotalin is a major derivative of bufadienolides which have a similar structure compared with diosgenin, the aglucone of dioscin. And gamabufotalin inhibited the phosphorylation of IKKβ^43^. Our previous studies also demonstrated blocking NF-κB pathway by dioscin to exert anti-inflammation and protective effect^44^,^45^. Therefore, dioscin might target IKK to inhibit IκB-α phosphorylation and thereby abrogate the activity of NF-κB.

### Dioscin induced autophagy via inhibition of PI3K/Akt in MCF-7and MCF-7/ADR cells

Due to the low level of MDR1, the observed sensitization effect of dioscin in MCF-7 cells could depend on a non-MDR1 mechanism. Indeed, the expression of MDR1 in MCF-7 cells was under the limit of detection in our previous study^31^. But we found induction of autophagy by dioscin in both MCF-7 and MCF-7/ADR cells (Fig. 4a,b). Autophagy is a cellular process by which cytoplasmic material is either degraded to maintain homeostasis or recycled for energy and nutrients in starvation. The role of autophagy in tumors is complex and controversial due to its potential to either induce cell death or promote cell survival^14^. The dual role of autophagy makes it to be a potential therapeutic target of many diseases such as infections, neurodegeneration, aging, Crohn’s disease, heart disease and cancer^46^. Recently, autophagy has been demonstrated as a new reversal strategy in MDR cancer therapy. Activation of autophagy by voacamine could overcome drug resistance by inducing apoptosis-independent autophagic cell death^18^. Likewise, the autophagy inhibition by pterostilbene reversed MDR by sensitizing the cells to anticancer molecules^15^. In present study, autophagy induced by dioscin at IC10 enhanced the ADR chemosensitivity of MCF-7 and MCF-7/ADR cells and played a prodeath role in ADR treated MCF-7 and MCF-7/ADR cells (Fig. 5). At higher dose of dioscin, dioscin-induced autophagy was earlier than dioscin-induced apoptosis and provided a protective mechanism for cell survival^15^. Considering the dual role, autophagy may switch its prosurvival role to a prodeath one in different conditions. The regulation of autophagy is mediated by PI3K/Akt/mTOR signaling pathway and MEK1/2/ ERK1/2 pathway^14^,^17^. Our results showed that dioscin induced autophagy by the inhibition of PI3K/Akt phosphorylation (Fig. 6) but did not block the phosphorylation of ERK in both MCF-7 and MCF-7/ADR cells (data not shown). The blockade of PI3K/Akt pathway by dioscin was also reported by other research^47^,^48^. The PI3K/Akt signaling pathway is a major driving force in a variety of cellular functions and dysregulation of PI3K/Akt has been implicated in many human diseases including cancer^49^. Moreover, PI3K/Akt pathway was thought to regulate the expression of MDR1 and PI3K/Akt inhibition correlated down-regulation of NF-κB activity and inhibition Pgp function^50^,^51^. Our previous study demonstrated that downregulating P-gp expression derived from resveratrol was mediated by suppressing the PI3K/Akt/mTOR signaling pathway in K562/ADR cells^52^. We noted that the autophagy level was higher and PI3K/AKT activity was lower in MCF7/ADR cells (Fig. 6). A higher level of autophagy induced by voacamine was found in multidrug resistant human osteosarcoma cells and the exact mechanism was not clearly understood^18^. Kam *et al*. thought that resistance to therapy requires energy and other resources which are, thus, diverted from proliferation and invasion^53^. A higher level of autophagy in MDR cells might represent the fitness cost of their resistance mechanisms. In terms of our results, dioscin-induced autophagy enhanced the ADR chemosensitivity of MCF-7 and MCF-7/ADR cells via inhibition of PI3K/Akt pathway. Therefore, the sensibilization effect of dioscin in MCF-7/ADR cells was related to multiple pathways including at least NF-κB and PI3K/Akt pathway.

To date, strategies to overcome resistance are more and more popular with the researchers concerned. Considering the roles of miRNA and lncRNA in breast cancer progression and the development of endocrine resistance^24^, siRNA therapeutics using targeting delivery materials could be a potential strategy to overcome endocrine resistance^54^. Use of combination anti-HER2 treatments for potent inhibition of the HER family signaling is biologically sound and offers great clinical promise for breast cancer with HER-2 targeted therapy resistance^23^. In present study, we found that dioscin increased the activity of ADR in MCF-7 and MCF-7/ADR cells through downregulation of MDR1 and induction of autophagy. While modulation of drug efflux bump is well established strategy to overcome multidrug resistance, role of autophagy induction in MDR reversal was now existence controversy. Based on our results, autophagy induction by dioscin enhanced the apoptosis induced by ADR in both sensitive and resistant MCF-7 cells, providing a new insight into the potential use of dioscin in the treatment of breast cancer with MDR.

In summary, our data strongly imply that dioscin (1) can increase the activity of ADR in MCF-7 and MCF-7/ADR cells at nontoxic concentrations, (2) works by down-regulating MDR1 by a mechanism involving the inhibition of the NF-κB signaling pathway in MCF-7/ADR cells, and (3) works by activation of autophagy via inhibition of PI3K/Akt pathway in both MCF-7 and MCF-7/ADR cells. Dioscin is potentially a novel, potent and clinically relevant MDR reversal agent. These findings provide evidence in support of further investigation into the clinical application of dioscin as a chemotherapy adjuvant.

## Methods

### Materials

Dioscin was kindly provided by Professor Jinyong Peng (College of Pharmacy, Dalian Medical University, Dalian, China). The purity of dioscin was 96.55% as determined by HPLC^55^. ADR was from Shenzhen Main Luck Pharmaceuticals, Inc. (Shenzhen, China). DMEM was purchased from Gibco BRL (Grand Island, NY, USA). Fetal bovine serum (FBS) was from Invitrogen Life Technologies Corporation (Invitrogen, Carlsband, CA, USA). TNF-α was purchased from Peprotech (Rocky Hill, NJ, USA). 3-MA, LY294002, antibodies against LC3-I/II, Akt, Phospho-Akt, PI3K, Phospho-PI3K phospho-IκB-α and horseradish peroxidase-conjugated anti-mouse IgG antibodies were obtained from Cell Signaling Technology (Beverly, MA, USA). Antibodies against β-actin and MDR1 were purchased from Santa Cruz Biotechnology (Santa Cruz, CA, USA). 3-(4, 5-dimethylthiazol-2-yl)-2, 5-diphenyltetrazolium bromide (MTT) was from USB Corporation (Cleveland, OH, USA). All other reagents and solvents were of analytical grade.

### Cell culture

MCF-7 and MCF-7/ADR cells were purchased from Nanjing KeyGen Biotech Co., Ltd. (Nanjing, China) and were grown in RPMI 1640 medium supplemented with 10% FBS, 0.01 mg/ml bovine insulin, 100 units/mL penicillin, and 0.1 mg/mL streptomycin and kept in an incubator at 37 °C with 95% humidity and 5% CO_2_.

### Cytotoxicity Assays

Cell viability was determined using an MTT assay as described previously^12^. MCF-7 and MCF-7/ADR cells were trypsinized (0.25% Trypsin + 0.53 mM EDTA), disaggregated through a pipette and counted in a hemacytometer. Cells were seeded in 96-well plates (8000 cells/well) and then treated with various concentrations of ADR with or without dioscin. After incubation for 48 h, 10 μL of MTT reagent was added to each well and left to incubate for an additional 4 h. A 100 μL aliquot of SDS-isobutanol-HCl solution was added and left to incubate overnight. Relative cell viability was obtained on a microplate reader (Bio-Rad, San Diego, CA, USA) with a 570 nm filter. IC50 values were calculated from a graph of percent proliferation vs inhibitor concentration using Prism (Graphpad Software, La Jolla, CA, USA).

### Morphological analysis

MCF-7 and MCF-7/ADR cells were treated with 0.4 μM dioscin for 24 h and observed with an Olympus CKX41 (Olympus, Tokyo, Japan).

### ADR accumulation assay

Cells were seeded in 24-well plates with a density of 5 × 10^5^ cells/well and cultured for 3d before accumulation assay. The medium was then replaced by fresh medium with or without dioscin (0.4 μM) or verapamil (20 μM) for another 24 h. Then cells were incubated with 10 μM ADR for 1 h. After three washes with ice-cold PBS, the cell monolayers were subsequently lysed and the concentration of ADR in cell lysate was determined by LC-MS/MS^56^.

### Annexin V/PI double staining

To detect apoptosis in MCF-7 and MCF-7/ADR cells after exposure to ADR, an FITC Annexin V Apoptosis Detection Kit (keyGEN BioTECH, Nanjing, China) was used to quantify cell numbers in different stages of cell death. After washed twice with ice cold PBS, 1 × 10^5^ cells were resuspended in 500 μl 1× binding buffer (0.01 M Hepes/NaOH (pH 7.4), 0.14 M NaCl, and 2.5 mM CaCl2). After addition of 5 μl FITC Annexin V and 5 μl PI, the cell suspension was gently mixed and then incubated for 15 min at room temperature in the dark, followed by flow cytometry analysis within 1 hour.

### Quantitative RT-PCR

Gene expression was determined by qRT-PCR as described previously^12^. The following oligonucleotides were used as primer sequences: 5′- GGAGCCTACTTGGTGGCACATAA-3′ (forward) and 5′-TGGCATAGTCAGGAGCAAATGAAC-3′ (reverse) for MDR1; 5′-CGCGAGAA ATGACCCAGAT-3′ (forward) and 5′-GTACGGCCAGAGGCGTACAG-3′ (reverse) for β-actin.

### Western Blotting

Protein expression was examined by western blot as described previously withom temperature in the dark, followed by flowor modifications^12^. PVDF membranes (Millipore, Billerica, MA, USA) were incubated overnight at 4 °C with 1:100, 1:500, 1:1000, 1:500, 1:1000, 1:2000 and 1:1000 dilutions of monoclonal antibodies for MDR1, LC3-I/II, Akt, Phospho-Akt, PI3K, Phospho-PI3K and Phospho-IκB-α (Ser32/36), respectively. A 1:2000 dilution of monoclonal antibody for β-actin was used. After incubation with a primary antibody, membranes were rinsed three times with TTBS and incubated with anti-mouse horseradish peroxidase-conjugated secondary antibodies for 2 h at 37 °C. After extensive washing with TTBS, membranes were exposed to enhanced chemiluminescence-plus reagents (ECL) from the Beyotime Institute of Biotechnology (Haimen, China) according to the manufacturer’s protocol. Emitted light was recorded with a BioSpectrum-410 multispectral imaging system using a Chemi HR camera 410. Protein bands were visualized and photographed under transmitted ultraviolet light. Images were used for semi-quantitative measurements based on band densitometry.

### Transient Transfection and Luciferase Assays

To determine promoter activity, a dual-luciferase reporter assay system (Promega, Madison, WI, USA) was used. Briefly, cells were plated in 24-well plates overnight and transiently co-transfected with hMDR1-Luc or NF-κB-Luc construct and pRL-SV plasmid (Renilla luciferase expression for normalization) (Promega, Madison, WI, USA) using LipofectAMINETM 2000 reagent (Invitrogen, Carlsbad, CA, USA). Cells were then exposed to dioscin and TNF-α for 48 h. Luciferase activity in cell lysates was measured using a TD-20 luminometer (Turner Designs, Sunnyvale, CA, USA). Relative luciferase activity was calculated by normalizing MDR1 or NF-κB promoter-driven firefly luciferase activity to Renilla luciferase activity (Luminoskan Ascent, Thermo Electron).

### Statistical analysis

All experiments were done in triplicate. The SPSS 19.0 statistical package was employed to perform correlation analysis. One-way analysis of variance (ANOVA) was used to determine the significance of difference s between treatment groups. The Fisher’s least significant difference (LSD) test was used for multigroup comparisons. Statistical significance was considered to be p < 0.05.

## Additional Information

**How to cite this article**: Wang, C. *et al*. Dioscin strengthens the efficiency of adriamycin in MCF-7 and MCF-7/ADR cells through autophagy induction: More than just down-regulation of MDR1. *Sci. Rep.* **6**, 28403; doi: 10.1038/srep28403 (2016).

## Supplementary Material

Supplementary Information
