# Supplementary material for: Dioscin strengthens the efficiency of adriamycin in MCF-7 and MCF-7/ADR cells through autophagy induction: More than just down-regulation of MDR1
Source: Sci Rep. 2016 Jun 22;6:28403. doi: 10.1038/srep28403 (PMC4916600; doi:10.1038/srep28403)
Supplement: Supplementary Information [file srep28403-s1.doc]

Supplementary data

**Dioscin strengthens the efficiency of adriamycin in MCF-7 and MCF-7/ADR cells through autophagy induction: More than just down-regulation of MDR1**

Changyuan Wang^1,2^, Xiaokui Huo^1,2^,Lijuan Wang^1^, Qiang Meng^1,2^, Zhihao Liu ^1,2^,Qi Liu^1,2^, Huijun Sun^1,2^, Pengyuan Sun^1,2^, Jinyong Peng^1,2^ & Kexin Liu^1,2,*^

**1.** **The** **cytotoxicity of dioscin and adriamycin (ADR) on MDA-MB-231 cells**

Cell viability of MDA-MB-231 was determined using an MTT assay. Both dioscin and ADR inhibited the proliferation of MDA-MB-231 cells in a concentration- and time-dependent manner (Fig. S1). The non-toxic concentrations of dioscin (namely IC10) were 0.26 μM (24h) and 0.18 μM (48h) (Fig. S1a). IC50 values of ADR were calculated as 0.64 μM and 0.24 μM after incubation of 24h and 48h (Fig. S1b), respectively. Moreover, the inhibitory effect was significantly enhanced and IC50 value of ADR was reduced to 0.10 μM when the medium contained dioscin at a final concentration of 0.20 μM (Fig. S1b). The result indicated dioscin even at non-toxic concentration potentiated the cytotoxic effect of ADR in MDA-MB-231 cells.


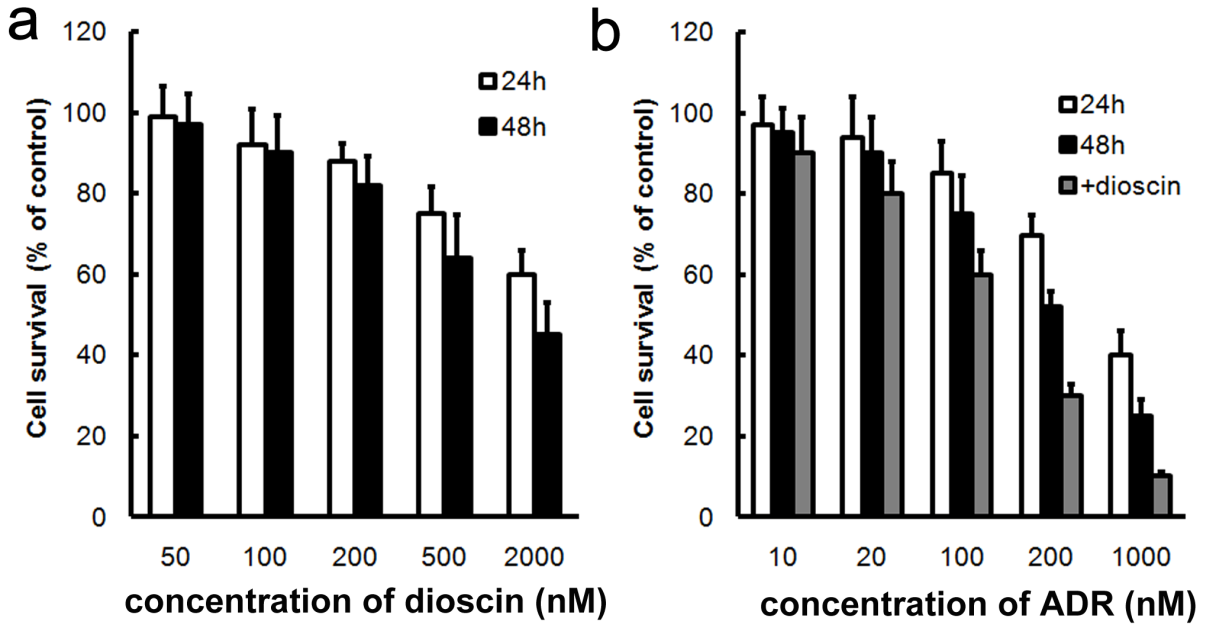


Fig. S1 Dioscin at nontoxic concentration increased ADR chemosensitivity in MDA-MB-231 cells. MDA-MB-231 cells were seeded in 96-well plates (4000 cells/well) and then treated with various concentrations of dioscin and/or ADR. a, cells were treated with dioscin (0.05-2 µM) for 24 or 48 h. b, cells were treated with ADR (0.01-1 µM) for 24 h or treated with ADR (0.01-1 µM) with or without dioscin (0.2 µM) for 48 h. The survival cells were then determined by MTT assay.

**2.** **Induction of autophagy by dioscin in MDA-MB-231 cells**

Autophagy induction by dioscin was evaluated though morphology observation and LC3-I-to-LC3-II conversion. Cytoplasmic vacuoles were observed after 24-h treatment with dioscin (0.2 µM) in MDA-MB-231 cells (Fig. S1a), suggesting that autophagy was induced by dioscin. The induction was then confirmed by western blot assays. Dioscin incubation initiated the transformation from LC3-I to LC3-II and increased the protein level of beclin-1 (Fig. S1a), which were the commonly used autophagy makers. These results suggested that dioscin induced autophagy in MDA-MB-231 cells.


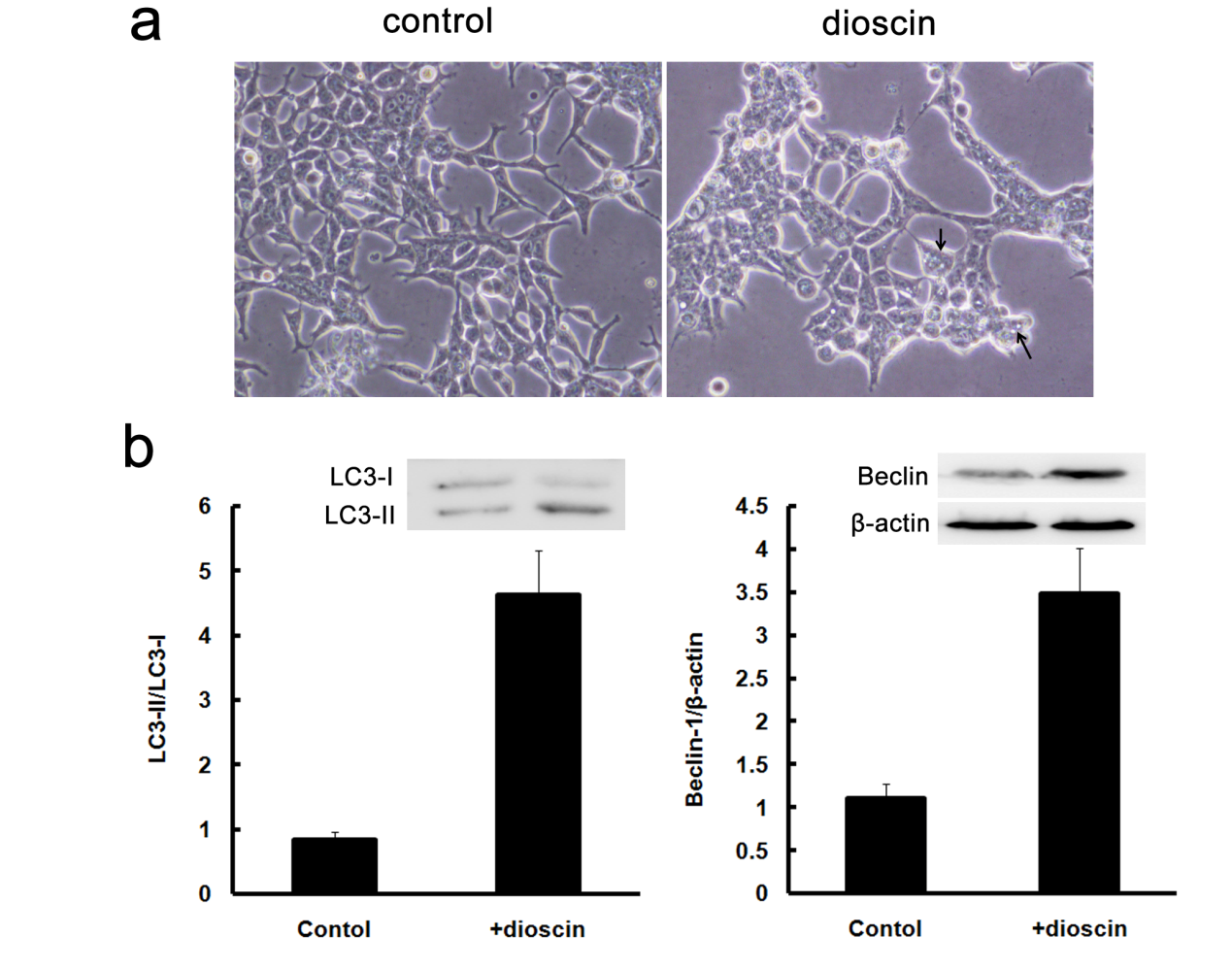


Fig. S2 Dioscin induced autophagy in MDA-MB-231 cells. a, Cells were treated with 0.4 μM dioscin for 24 h and cellular morphology was observed by phase-contrast microscopy. Typical vacuoles in the cytoplasm were marked by arrowhead. Magnification: 400×. b, The cells were subsequently lysed and the total protein were extracted for determination of LC3-I, LC3-II and beclin-1 protein expression by Western blotting.

**3.** **Induction of autophagy by dioscin accelerated apoptosis of MDA-MB-231 cells**

The role of autophagy induction on ADR cytotoxicity was further investigated through MTT and apoptosis assays in the absence or presence of autophagy inhibitor 3-MA. Incubation with dioscin (0.2 μM) or 3-MA (5 mM) alone almost unchanged MDA-MB-231 cell survival, however, co-incubation of dioscin enhanced the cytotoxic effect of ADR in ADR+dioscin group and the sensitization effect of dioscin was partially abolished by 3-MA in ADR+dioscin+3-MA group (Fig. S3a). The results of Annexin V/PI double staining further confirmed that inhibition of autophagy weakened the sensitization effect of dioscin. The fraction of apoptotic cells induced by ADR was significantly increased by dioscin in MDA-MB-231 cells (Fig. S3b). Similarly, 3-MA counteracted the effect of dioscin on ADR cytotoxicity (Fig. S3b). These results indicated that dioscin induced autophagy and increased ADR cytotoxicity in MDA-MB-231.


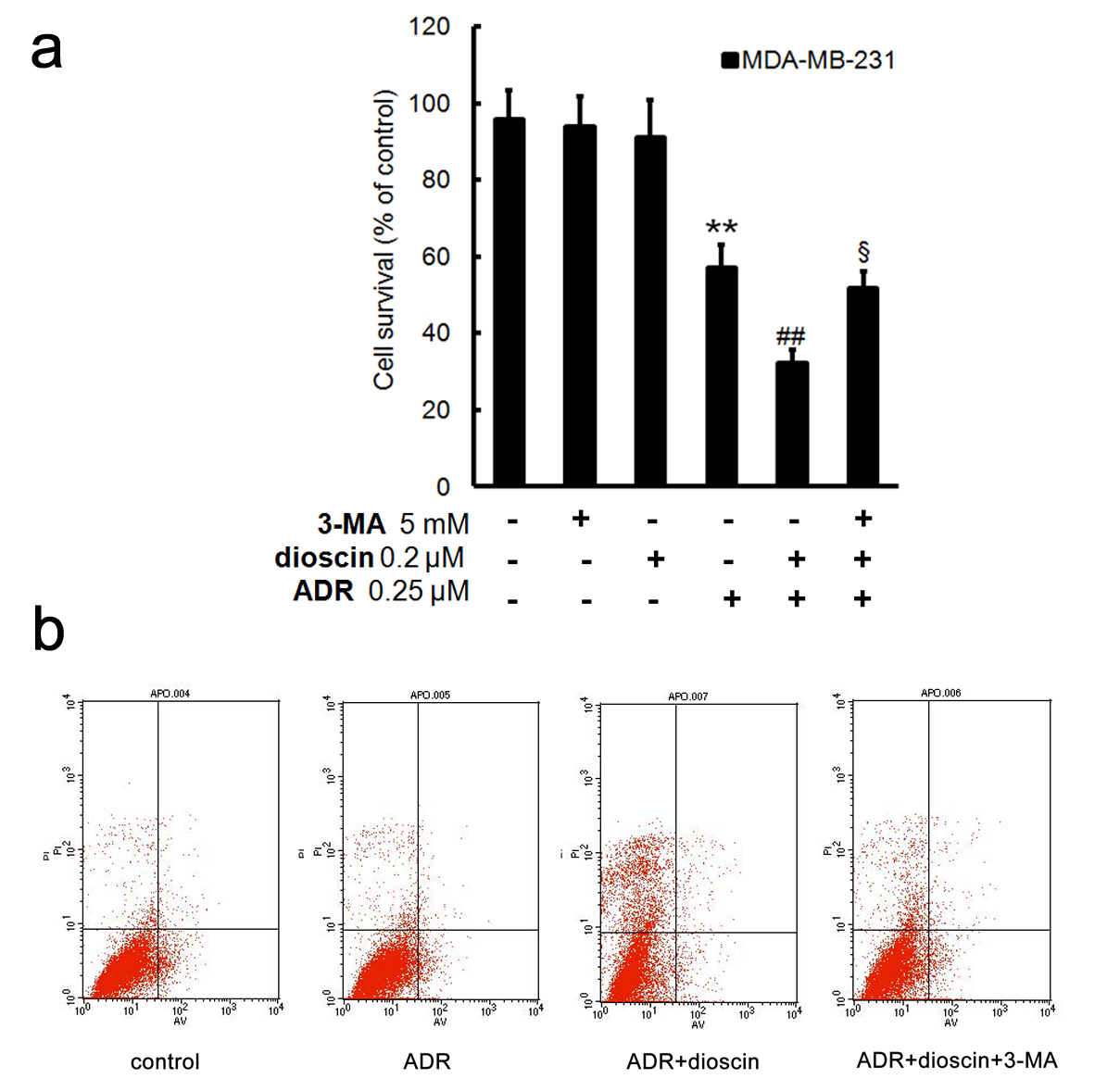


**Fig. S3** Dioscin-induced autophagy accelerated cellular death in MDA-MB-231 cells. A, cells were treated with 3-MA, dioscin and/or ADR for 24h and cell viability was determined by MTT assay (a) or Annexin V/PI double staining (b). **p < 0.01 versus that obtained in the control group. ##p < 0.01 versus that obtained in ADR alone group. §p < 0.05 versus that obtained in the ADR + dioscin group.

**4.** **Dioscin induced autophagy in MDA-MB-231 cells via inhibition of PI3K/Akt pathway**

The role of PI3K/Akt pathway in the autophagy induction by dioscin was evaluated by determining the expression levels of PI3K/Akt and LC3-I/ LC3-II with or without PI3K inhibitor LY294002 in MDA-MB-231 cells. Dioscin treatment decreased the levels of phosphorylated PI3K and Akt (Fig. S4a and S4b) in MDA-MB-231 cells. Moreover, the levels of LC3-II were up-regulated by dioscin as well as LY294002, an inhibitor of PI3K/Akt pathway (Fig. S4c). Taken together, dioscin-induced autophagy is mediated by inhibition of PI3K/AKT in MDA-MB-231 cells.


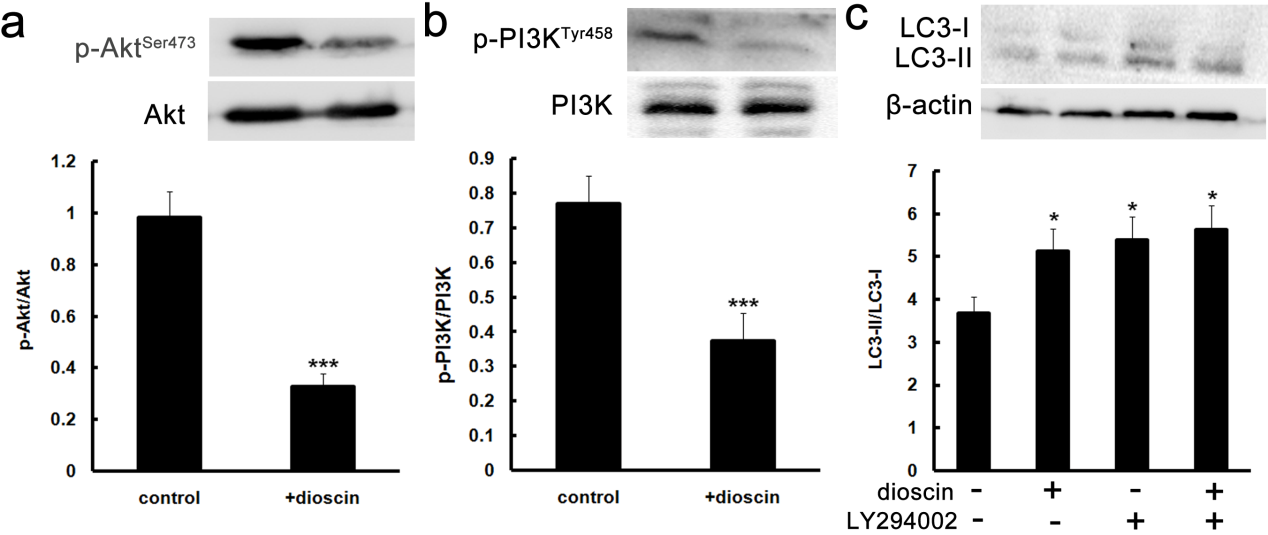


**Fig. S3** Dioscin induced autophagy through inhibition of PI3K/AKT pathways in MDA-MB-231 cells. Cells were treated with dioscin (0.2 µM) with or without PI3K/AKT inhibitor LY294002 for 24 h and the levels of phosphorylation of Akt (a) and PI3K (b), and LC3-I/ LC3-II in MDA-MB-231 cells. ***p < 0.001, **p < 0.01 versus that obtained in the corresponding control group.
